# Immunological network signatures of cancer progression and survival

**DOI:** 10.1186/1755-8794-4-28

**Published:** 2011-03-31

**Authors:** Trevor Clancy, Marco Pedicini, Filippo Castiglione, Daniele Santoni, Vegard Nygaard, Timothy J Lavelle, Mikael Benson, Eivind Hovig

**Affiliations:** 1 Department of Tumor Biology, Institute for Cancer Research, The Norwegian Radium Hospital, Oslo University Hospital, Oslo, Norway; 2 Institute for Computing Applications, National Research Council, Rome, Italy; 3 The Unit for Clinical Systems Biology, University of Gothenburg, Gothenburg, Sweden; 4 Institute of Medical Informatics, The Norwegian Radium Hospital, Oslo University Hospital, Oslo, Norway; 5 Department of Informatics, The University of Oslo, Oslo, Norway

## Abstract

**Background:**

The immune contribution to cancer progression is complex and difficult to characterize. For example in tumors, immune gene expression is detected from the combination of normal, tumor and immune cells in the tumor microenvironment. Profiling the immune component of tumors may facilitate the characterization of the poorly understood roles immunity plays in cancer progression. However, the current approaches to analyze the immune component of a tumor rely on incomplete identification of immune factors.

**Methods:**

To facilitate a more comprehensive approach, we created a ranked immunological relevance score for all human genes, developed using a novel strategy that combines text mining and information theory. We used this score to assign an immunological grade to gene expression profiles, and thereby quantify the immunological component of tumors. This immunological relevance score was benchmarked against existing manually curated immune resources as well as high-throughput studies. To further characterize immunological relevance for genes, the relevance score was charted against both the human interactome and cancer information, forming an expanded interactome landscape of tumor immunity. We applied this approach to expression profiles in melanomas, thus identifying and grading their immunological components, followed by identification of their associated protein interactions.

**Results:**

The power of this strategy was demonstrated by the observation of early activation of the adaptive immune response and the diversity of the immune component during melanoma progression. Furthermore, the genome-wide immunological relevance score classified melanoma patient groups, whose immunological grade correlated with clinical features, such as immune phenotypes and survival.

**Conclusions:**

The assignment of a ranked immunological relevance score to all human genes extends the content of existing immune gene resources and enriches our understanding of immune involvement in complex biological networks. The application of this approach to tumor immunity represents an automated systems strategy that quantifies the immunological component in complex disease. In so doing, it stratifies patients according to their immune profiles, which may lead to effective computational prognostic and clinical guides.

## Background

Although a link between the immunity and cancer was observed almost 150 years ago [1], the exact nature of the relationship has been developed and debated through several stages of complexity. In recent years, it has been established that the immune system plays crucial roles in tumor development [2], and indeed on patient survival for various cancers [3-7]. Due to a lack of comprehensive analytical approaches, molecular characterization of the roles of the tumor immune component has been somewhat difficult to elucidate on a genome-wide scale.

Current strategies to identify the immune component of tumors tend to employ incomplete manual efforts that do not grade the immune genes. Indeed, even the very definition of an immune gene is unclear, as several interconnected subsystems comprise the totality of immunity. In addition, an analysis of the molecular interactions linked to tumor immunity is usually limited to a pathway-centric paradigm, which is often hindered by the complexity in which immune pathways are entangled in signaling crosstalk [8]. These challenges are further complicated during cancer progression by the migration of immune cells into unique microenvironments, and by the altered expression of immune genes intrinsic to the tumor. Consequently, as in a tumor gene expression profile, it is not trivial to grade the immune component or identify its related molecular networks.

Multidisciplinary and integrated strategies that handle these and other complex challenges of tumor immunity are increasingly sought after [9-16]. With recent advances in genomics, and increased amounts of latent detailed knowledge in the medical literature, computational approaches can now be developed to study the importance of immune genes and their networks of interactions linked to cancer progression.

Consequently, we have devised a strategy that assigns a ranked immunological relevance score to all human genes for the purpose of profiling the immune component of tumor gene expression. Coupling text mining to information theory, this approach charts immunological relevance onto the human interactome. To apply this strategy in a cancer specific manner, we analyzed melanomas. We first identified immunological signatures that were differentially regulated in the progression from primary stages of skin cancer through to metastases [17]. Survival data from a set of advanced stage melanoma patients were also analyzed, to assess the link between immunological relevance of genes in expression profiles and clinical outcome [5,18].

Our computational approach to assign immunological relevance to genes was benchmarked against manual efforts that identify immune genes, and the strategy was shown to substantiate the performance of existing immunological grading systems. Furthermore, it identified the ranked immunological components of the expression profile of a tumor with its associated networks of interactions. This informative grading of the magnitude of the immune component from patient gene expression profiles may serve as a computational diagnostic and prognostic guide to assess the aggressiveness of a given tumor.

## Results

### An information theoretical approach to assign immunological relevance to genes

A comprehensive list of 1921 immunology terms was compiled by manual selection of the most relevant terms from the standard biomedical vocabularies of Medical Subject Headings (MeSH) in Medline and the Gene Ontology (GO) controlled vocabulary (see Methods: "Defining the dictionary of terms for immune and neoplasm relevance"). This broad set of terms was collectively considered to be the immunological symbols of communication stored in the over 20 million articles of the biomedical literature (Additional file 1). Using established text mining procedures [19] (see Methods: "Extraction of human genes, immune and neoplasm terms from Medline"), we used these terms and their relationships to gene citations in Medline by capitalizing on the universal feature of coded *information*, present in all forms of communication. By this, it is implied that immune relevant genes have a level of immune information content quantified using this combined set of immune terms in Medline, which is greater than that of genes that play a lesser role in the immune system. Information theory calculations were used to measure the size of the immunological message stored for each human gene with respect to these terms. The probabilities in the information theory calculations were defined through the frequency by which a given gene is cited with a given immune term relative to the number of times the immune term is cited in Medline among all human genes with that term. This measure of immune information content for a gene may be biased by the higher frequency of certain genes being associated overall with the sources of the immune terms, *i.e. *the popularity of a gene among all terms in the biomedical vocabularies. This bias was corrected for using a method in information theory known as the Kullback-Leibler (KL) divergence (see Methods: "An immunological and cancer relevance score for all human genes using information theory and text mining"). The KL score for all human genes was defined as the "immunological relevance" for a gene and termed as such throughout this study (Additional file 2). A similar strategy was also applied to a manual selection of 562 cancer disease terms to determine a genome-wide cancer relevance score for every human gene.

### Benchmarking of the immunological relevance score and the extension of immune gene resources

**Figure 1:**
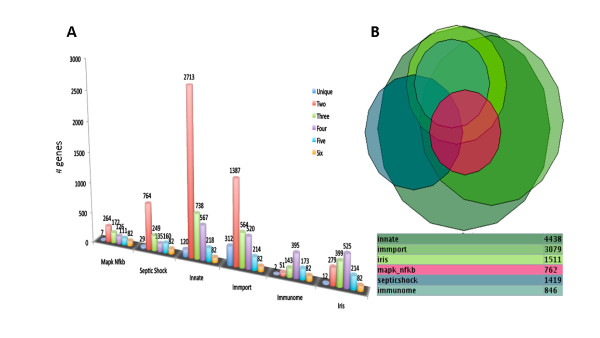
**Heterogeneous distribution of genes in immune databases and an incomplete catalogue of immune knowledge**. (**A**) Bar chart depicting the shared gene distribution of the immune resources. 82 of the total integrated set of 4833 genes are common to all 6 manually curate resources (orange colored bar). Few genes were unique to an individual database, ranging from a minimum of two for "Immunome" and 122 for the "Innate". (**B**) An approximation using a Venn Euler diagram illustrates the heterogeneous overlap among the different databases. The Innate database being the largest resource has the largest intersections. The septic shock resource has smaller overlaps with the others (with the exception of Innate) highlighting its focus on collating genes related to the response to bacterial toxins during septic shock.

**Figure 2:**
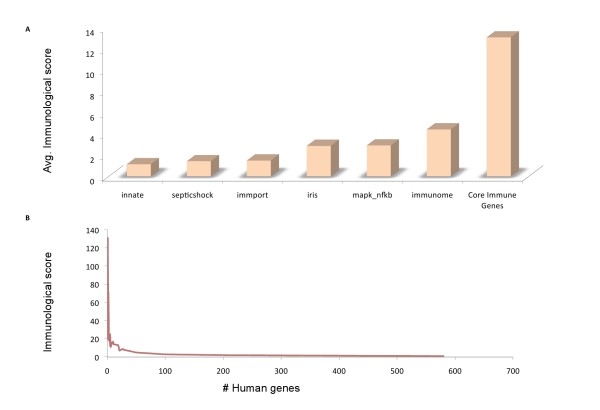
**Benchmarking of immunological relevance scores against manually curated immune resources**. **(A) **The mean immune score for each database is depicted in the bar chart. The core immune genes are those 82 genes that are common to all immune resources and have a significantly larger amount of information content in comparison to each of the individual immune resources. (**B**) The frequency distribution of all HUGO name assigned genes reveals a sharp decline in immune relevance across the genome.

In order to benchmark this immunological relevance for genes, we compared the score against a set of validated immune resources. We utilized gene sets from six manually curated immune efforts (see Methods: "Collating manually curated immune relevant gene sets") that contain independently annotated genes relevant for various aspects of immunity. There were a total of 4833 genes in this integrated set, which had a heterogeneous distribution across the six resources, in that only 82 core immune genes were common to all databases. Many genes in each resource were shared with merely one of the other resources, and few genes were unique to an individual resource (Figure 1). The benchmarking of the immunological relevance score against this set of manually curated immune resources is presented in Figure 2A. The average immunological relevance score over all genes in each database was determined, compared against each other and the genes not manually curated by these resources. The Immunome [20] ranked the highest among the six manually curated resources in terms of immune information content, reflecting its focus on collating genes enacting functions specific to immune cells. When measuring the immunological relevance of all genes assigned a name by the Human Genome Organization (HUGO) and not catalogued in any of the immune resources, the average approaches zero. The frequency distribution of immunological relevance for all human genes assigned a name in HUGO shows a sharp decline from high to low immunological relevance (Figure 2B), revealing distinct categories of immune and non-immune genes. Moreover, the top ranked genes in the non-curated list represent novel candidates for entry in immune resources (Additional file 3). To assess further the benefit of assigning an automatic immunological relevance score to genes, the integrated set of manually curated genes was compared against two large scale studies that have characterized the human inflammatory response: (1) the *endotoxin response network *from gene expression profiling in human leukocytes [21], and (2) the *inflammation assembly*, which consists of genes detected in genetic variants in inflammatory pathways [22]. The *endotoxin response network *and *inflammation assembly *had 66% and 13% non-overlapping genes with respect to the manually curated resources. The non-correspondence of these six expert resources with large-scale experimental efforts partly indicates the specialized nature of some of these resources and partly may indicate potential in further management of immune knowledge from expert curators. It may also illustrate that there could still be more genes to be implicated in human immunity that are as yet uncharted.

### The interactome landscape of immunological and cancer relevance

**Figure 3:**
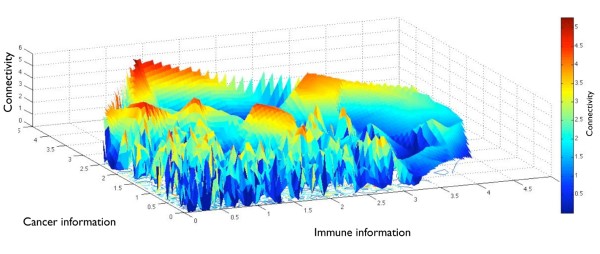
**The tumor-immunity interactome landscape**. A three-dimensional surface plot representing the landscape of degree centrality (connectivity) of the interactome in the context of immune and cancer relevance: All axes are on the log scale and values above one on the log scale were considered high in terms of immune and cancer relevance. The consideration of one on the log scale as high in terms of immune relevance is made on the basis of the average immune scores for the expert sources ranging from 1 bit and above (see Figure 2). The color scale in the heatmap is representative of the connectivity of each gene in the human interactome. That which is apparent is the distinct areas of scattered high and low connectivity for genes in the cancer-immune landscape. The underlying data for this plot is detailed in Additional File 5.

An affirmed realization from the post genomic era is that no gene functions in isolation, but rather is embedded in a complex network of interacting molecules [23]. Our strategy to profile the immune component of tumors would therefore benefit from an analysis of how immunological relevance relates to the position a gene occupies in complex cellular networks (in this case an integration of three human interactome databases, see Methods: "Constructing a validated human interactome & network analysis"). The creation of a validated and ranked score of a gene's immunological relevance allowed us to chart this score in a landscape setting against cancer relevance and the positional importance (*centrality*) of a gene in the interactome (Figure 3). Centrality is a class of network measurements used to determine the relative importance of a gene in cellular networks. We analyzed five different centrality measures the principal of which being connectivity (i.e. number of interactions per gene). Genes from the six manually curated immune resources on average had a higher connectivity relative the entire interactome (data not shown). Interestingly, increasing immunological or cancer relevance showed no strong correlation with connectivity or to any of the four other network centrality measures (Additional file 4). The immune and cancer genes harboring the highest connectivity (network hubs) raise the average, and were unevenly distributed across the heterogeneous interactome landscape (Figure 3). This analysis allowed the detection of scattered peak regions whose genes play driver roles in propagating signals with importance to tumor-immune crosstalk. The classical coordinator of tumor-immune interactions, *IFNG*, and various T-cell markers were among the highest ranked in this high peak category, as displayed in the underlying interactome landscape data in Additional file 5. We also observed a high degree of correlation (0.75 Spearman's coefficient) between immunological and cancer information content across the genome.

### Immunological comparisons of normal tissues and robustness of tissue specific immune interactions

**Figure 4:**
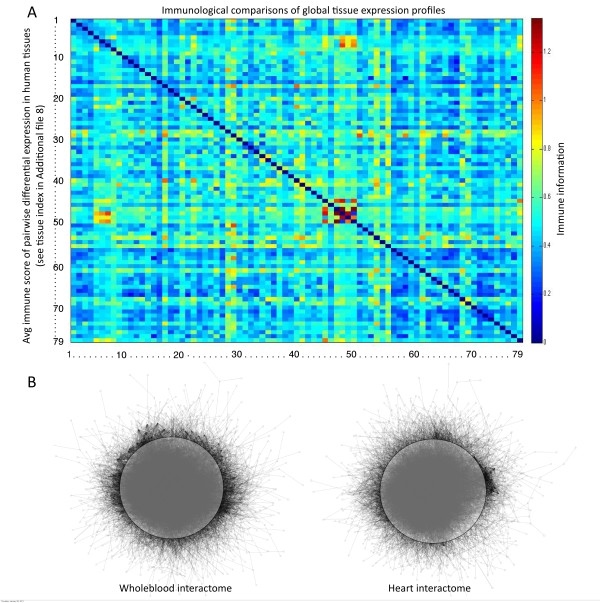
**Immunological components of normal tissue**. **(A) **Heatmap of the immunological gene expression fold-change comparisons among the 79 tissues from the SymAtlas [24]. This matrix displays the average immune score from those genes that contribute to greater than 2 times fold change difference between each tissue's pairwise comparisons. This combination of expression profiling and immunological grading detects a heterogeneous difference in the immunological components between tissues in a global manner. Both the X and Y-axis are numerical index of the 79 tissues (the mapping of this index to tissue name is listed in Additional File 8). With respect to the robustness of immune genes in the interactome : **(B) **Tissue specific interactome networks for Wholeblood (eccentricity centrality = 0.67) and Heart (eccentricity centrality = 0.72). The difference in the average eccentricity value is only marginally visible by eye as evidenced by a lower symmetry of the Heart network (the same transparent circle drawn on top of the two networks displayed by means of the same algorithm using the software yEd).

As gene expression profiles of both normal and tumor tissue represent the combined signal of all cell types present in a sample: a global evaluation of the immunological component of normal tissue profiles was attempted, prior to the particular goal of quantifying such for tumors. For this purpose, we calculated the pairwise fold change comparisons of the differentially expressed genes among the 79 tissues profiles from the SymAtlas project [24] and the average immunological relevance score for those differentially expressed genes (shown in heatmap Figure 4A). A gene was considered differentially expressed if it had greater than a two-fold difference in expression between the two tissues under comparison. The pairwise comparisons revealed heterogeneous differences in immunological components among normal tissues (see heatmap in Figure 4A). The comparison, for example, between CD4 and CD8 positive T-cells from the tissue SymAtlas [24] had the largest immunological difference (see heatmap in Figure 4A, columns No 48 and 50 for CD4 and CD8 respectively). The procedure used to determine the differentially expressed genes is detailed in the Methods section entitled: "Microarray gene expression analysis and a composite expression and immunological relevance score".

**Figure 5:**
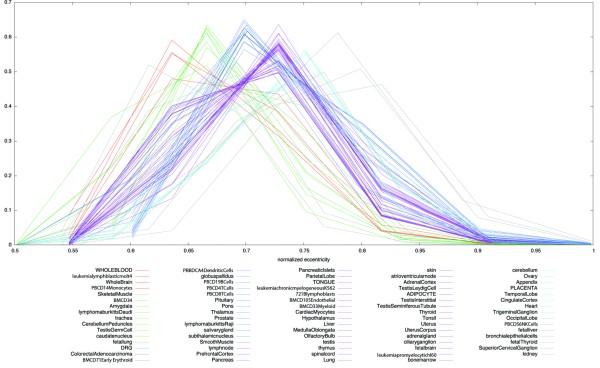
**Normalized frequency distribution of tissue specific eccentricity**. The distribution (*i.e.*, normalized frequency) of the gene eccentricity centralities for each of the tissue specific interactome networks (the same 79 human tissues profiled in Figure 4). Different network groups can be classified on the basis of the maximal value of the eccentricity distribution. Some network groups have a differential maximal value of distribution, and immune cells had the lowest values. The lower eccentricity values of immune cells reinforce the postulate that immune genes have robust reach throughout the human interactome. Equal colors in the legend correspond to equal maximal values of the normalized eccentricity.

In order to characterize the differences in the immunological component of these tissues from the perspective of the interactome, we used tissue specific networks previously determined for the SymAtlas tissue profiles [25]. In addition to connectivity, we calculated four other network centrality measures on each of these tissues networks (betweeness, eigenvector, closeness and eccentricity). To test if any of these centrality measures is a discernible property more specific to immune cells, we implemented K-means clustering on all five of the centrality measures across the tissues. Eccentricity was the only measure that classified the tissues in a biological meaningful manner (with K = 9 clusters), in that closely related tissues clustered together (e.g. neurological or immune related tissues, see cluster groups in Additional file 6). Moreover the distribution of the gene eccentricity centralities for each of the tissue interactome networks showed that immune cells had the lowest average eccentricity values (see brown lines, peak value at 0.63 in Figure 5). Leukocytes clustered into three classes, with CD4 and CD8 positive T-cells grouped with wholeblood, lymphoblast precursors into their own separate class, and the remainder of the blood cells profiled (including dendritic cells and NK cells) into a third class (Figure 5 and cluster groups in Additional file 6). The interaction network of a tissue (wholeblood) from the former immune cluster was significantly different from a random network (Wilcox rank, p-val of 0.01) and illustrated graphically in a comparison of this network to that of a non-immune tissue (heart) in Figure 4B. The difference in average eccentricity values between these two tissues is marginally visible in Figure 4B. As immune cells express more immune relevant genes and their eccentricity measures relate to shorter network distance, overall this tissue group clustering suggests that immune genes have more robust connections in the interactome.

### Immunological networks signatures and clinical outcome from expression profiles in melanoma patients

**Figure 6:**
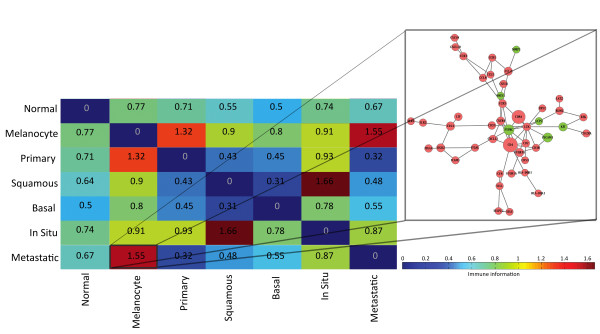
**Comparison of the immunological component of skin cancer and states of melanoma progression**. A heatmap of the average bits of immune information of the differentially expressed genes (> 2 times fold-change) among the pairwise comparisons of normal skin and skin cancer states. The labels from the left to right columns refer to normal skin tissues: ("Normal"), normal melanocyte ("Melanocyte") and then various states of skin cancer: primary melanoma ("Primary"), squamous cell carcinoma ("Squamous"), basal cell carcinoma ("Basal"), in-situ melanoma ("In Situ") and metastatic melanoma ("Metastatic"). Distinct differences in the immunological component of the various skin cancer and normal states are detected. We have focused here as an example, on the comparison between metastatic melanoma and normal human melanocytes. A subnetwork module from the interactome landscape of those genes with high immunological relevance is displayed. Upregulated genes are color-coded red and downregulated genes are color-coded green in this network. The size of a gene is proportional to the immunological relevance of the gene. There is clearly increased T-cell activity such as the presence of increased expression of *CD8, CD4 *and *CD3 *T-cell markers. This coincides with upregulation of key chemokine and cytokine interactions.

We next extended the principle of tissue expression profiling of immunological signatures in normal tissue to that of expression of normal skin, primary skin tumors and metastatic melanoma [17]. From the pairwise comparison of genes with a greater than two-fold change in expression across these different tissue states, we averaged the immunological score for those genes differentially expressed (> 2 times fold change) and examined how this score differed across the various expression profiles (see Figure 6). Using this average immunological score, we detected clear differences in the stages of progression and related these comparisons to their immune subnetworks from the interactome (see Figure 6). There was a particularly high immunological difference between normal melanocytes and both metastatic and primary melanoma and between normal skin and both metastatic and primary melanoma. The magnitude of the immune component difference between metastatic and normal melanocytes is depicted in Figure 6, along with a related immunological subnetwork of interactions. This network signature shows strong T-cell activation as well as diverse tumor associated chemokine and cytokine activity. There was, however, a much smaller immunological difference between metastatic melanomas and primary melanoma compared to that of normal melanocytes (Figure 6). This suggested that the framework could detect putative signatures of adaptive immunity in mediating transitions at early stages of progression in these patients. The observation that the highest ranked immune genes in these comparisons, *CD4 *and *CD8*, were upregulated in primary melanoma and metastasis compared to normal melanocytes signified early and enduring T-cell infiltration. In this comparison, immunological scoring also prioritized markers of innate immune cells such as *PECAM *and *CD14 *among others, accompanied by cytokines of inflammatory responses (*IL15, IL7, IL18*, *IL1A, IL8*). Interestingly, there was also high ranking of an early Th2 tumor-promoting environment demonstrated by presence of the *IL13RA2 *gene and the Th1 inhibiting cytokine *IL10*. The smaller amount of immunological information captured in the comparison of primary to metastatic melanoma (Figure 6) was attributable not to high scoring leukocyte or inflammation markers, but by upregulation of immunogenic melanoma antigens (*MAGEA2/3*) and downregulation of apoptosis inducing *S100A8/9 *cytokines. Summarized gene lists of the top ranked immunological transitions of normal skin, primary and metastatic melanomas are presented in Table 1. In-situ melanoma (MIS) compared to squamous cell carcinoma (SCC) held the highest immunological difference among all the state comparisons (Figure 6). Some of the top ranked immune genes in that comparison included upregulation in SCC relative to MIS of the chemokine *CXCL13 *and downregulation of the innate immune gene *LTF*

**Table 1:** Top ranked immunological transitions of melanoma progression

Gene comparison conditions	Highest graded immune genes	Significance to Melanoma progression
Upregulated (> 2fc) in both primary and metastatic melanoma compared to normal melanocyte (Immunological relevance score for each gene (KL) > 11 bits).	CD4, IL10, CD8A, CD40, IL15, IL7, IL18, TNFSF13B, PTPRC, IL13RA2, IL1A, PECAM1, C5AR1, CD86, ISG20, IL18R1, CD14, ITGB2, ADORA3, FCGR3A, CCL2, IL8, CCR5, FCGR3B	Signatures of T-cell infiltration, T-cell activation and the inflammatory response. Inclusive of the Th1 inhibiting cytokines
Downregulated (> 2fc) in both primary and metastatic melanoma compared to normal melanocyte (Immunological relevance score for each gene (KL) > 0.5 bits).	MME, IL24, DPP4, CYGB, MSC, SLC7A8	Regulation of extracellular matrix (ECM) remodeling, through proteolytic enzymes, and amino acid transporters
Upregulated (> 2fc) in primary melanoma compared to normal melanocyte. Not subject to >2fc in metastasis (Immunological relevance score for each gene (KL) > 2 bits).	IL5, TNF, IL1RN, DARC, HLA-DRB4, CFP, PTPN6, CD1B, ELA2, IL17B, ATP8A2, SLPI, CD27, STAT4, CDA, IL26, DEFB4, NFKBIA, HRH1, XCL1, DEFB1, PDPN, CTSG, SDC1, GATA3, MSMB, CD24, POU1F1, PRDM1, EBF1	Cytokine activity that is pro-survival and towards ECM remodeling. Increased transcriptional activity related to T-cell activation in the primary tumor. Increased presence of MHC class II markers.
Downregulated (> 2fc) in primary melanoma compared to normal melanocyte. Not subject to >2fc in metastasis (Immunological relevance score for each gene (KL) > 1 bit).	BAX, TNFRSF10B, SV2A	Down-regulation is indicative of p53 dysfunction and transduction of apoptosis signals. Overall leading to pro-survival in the primary tumor compared to normal cells
Upregulated (> 2fc) in metastatic melanoma compared to normal melanocyte. Not subject to >2fc in primary. (Immunological relevance score for each gene (KL) > 1 bit).	CCRL2, HLA-DRB1, MDK, C4A, CD55, CD80, FCGR1A, KLRC4, ICAM1, SPI1, HCST, PPBP, FCGR2C, GPR160, CXCL16, FOS, SERPINA1	Mediators of inflammation, angiogenesis, cell growth, and cell migration. Also present are signals of humoral immunity in the form of T-cell activation and B-cell development genes
Downregulated (> 2fc) in metastatic melanoma compared to normal melanocyte. Not subject to >2fc in primary. (Immunological relevance score for each gene (KL) > 1 bit).	KIT, IRF4, MLANA, MMP1	Down regulation of cell adhesion, differentiation factors and regulators of the innate and adaptive immune systems. Possibly promoting the metastatic phenotype
Upregulated (> 2fc) in metastatic melanoma compared to primary (Immunological relevance score for each gene (KL) < 1 bit).	MAGEA3, CSAG2, MAGEA2, GAGE1, MAGEA12, GAGE3, FKBP10	Eliciting immune T cell activation in metastatic tumors, as a consequence of being expressed particularly in the metastatic stages, while having very restricted expression in normal cells
Downregulated (> 2fc) in metastatic melanoma compared to primary (Immunological relevance score for each gene (KL) > 1 bit).	S100A9, S100A8, SLPI, DEFB4, DEFB1, MSMB, CD24, DEFB103A, COL17A1	Altered matrix remodeling and migratory behavior. Dynamic changes in the (ECM) in the metastatic tumors. Inclusive in this is the down regulation of important chemoattractants of innate immune cells

Comparison of progressive melanoma states and their highest weighted immunological relevant genes

A composite gene expression and immunological relevance score was used to grade each patient expression profile and find clinical trends to immunological gene signatures (see Methods: "Microarray gene expression analysis and a composite expression and immunological relevance score"). Although the Riker *et al. *study was not accompanied by clinical outcome data, there was a trend in two patients with giant primary melanomas (Breslow thickness of 90 mm) and downregulation of highly relevant immunological genes (p-val, 0.02) compared to 12 other patients with primary melanomas. Using this composite grade, we examined the immunological differences in the outcome, as well as in other clinical features of 57 patients that had reached metastatic melanoma at stage IV [18] and 38 patients at stage III (Bogunovic et at, 2009). Notably, there was a significant association (p-val, 0) with the "high-immune" group of patients as annotated by Jonsson *et al *(as identified by one term, chosen a-priori). Similarly, the strategy detected downregulated highly relevant immunological genes in the patient group that fell into the "proliferative" group of patients (p-val, 0). An upregulated immunological trend was detected in patients that had favorable survival (p-val, 0.1) and was more significant (p-val, 0.02) in those patients categorized with "brisk" immune phenotype (infiltration of CD3 positive lymphocytes). The patient group with NRAS mutations (Q61L) had a correlation with downregulated immunological signatures (p-val, 0.007), hence classifying a group of patients with immune signaling interactions acting downstream of this oncogenic mutation. Patients with hypermethylation of the p16^INK4A ^promoter had trends towards upregulation of genes with high immunological relevance (p-val, 0.05). Overall, the trends with immunological grading of these expression profiles indicated that the assignment of an immunological relevance to genes could classify patient groups with varied immunological signatures. The same analysis was applied to 38 patients from (Bogunovic et al, 2009), and it revealed a significant correlation of upregulated immunological signatures in patients with prolonged survival (p-val, 0.0086) and a significant correlation of downregulated gene with patients that died (p-val, 0.0074). This was also the case in Jonsson et al, where each patient had a unique profile of clinical annotations and immunological gene expression levels (Additional file 7). Interestingly, the authors reported positive correlation with tumor infiltrating leukocytes (TILs) in those patients with favorable survival. A summary of these trends with patient clinical annotations and the immunological profiles for each patient is listed in Additional file 7.

## Discussion

The overlap between cancer and immunity has become increasingly well established in recent years. Epidemiologically, 15-20% of cancer deaths are associated to inflammatory conditions [26]. Furthermore, inflammation is a predisposition to cancer, and polymorphisms in cytokine genes are associated to cancer severity [27,28]. Although there is compelling evidence that supports this overlap, an understanding of the molecular mechanisms of what constitutes tumor-immune relationships is far from comprehensive [2,29]. This problem is complicated further by the uniqueness of the microenvironment of each tumor, and the complex interplay between cancer cell immune factors and immune cells infiltrating the tumor.

Gene expression profiling has the potential to provide an improved understanding of these complex relationships and address these challenges. Current approaches to assess the immune component of expression profiles are dependent upon the application of limited pre-defined sets of immune genes or terms. Prerequisite to the success of manual approaches is the challenge of defining the complete set of immune genes. We have demonstrated that this challenge has not been met. The crux in overcoming this challenge lies in what may be considered to be an immune relevant gene. One option to find immune genes with a role in cancer development is the use of expertly annotated databases [20,30-32]. Our approach improves on the limitations of manual approaches by applying a novel automated procedure that quantifies the immunological relevance for all human genes in bits of information. This score can be directly applied to and provide a more informative and quantitative assessment of the tumor immune component from the gene expression profile. The novel use of information bits to quantify the immunological component may be even further generalized, and applied to any phenotype or any other entity having been assigned symbols of written communication.

Having access to a ranked immunological relevance score for all genes provided an opportunity for analysis of the resulting interactome landscape for tumor immunity. This provided interesting insights into the relationships with levels of immune and cancer information of a gene in the interactome, in light of the new paradigm of network biology [23,33]. These observations in particular add to the debate of the importance of central positions held by cancer [34] and immune [35] genes in the cellular interactome network. Although there is on average higher connectivity for immune and cancer genes in those studies, we illustrated variation about the average, with certain peak genes raising the average connectivity in the interactome landscape.

Tissue specific expression analysis of the immunological relevance score demonstrated that there is a detectable difference among different tissues in the expression of immune genes. Tissue specific network analysis demonstrated that immune genes have distinguishably robust connections within a cells interactome. These observations may be explained by the diverse properties of various tissues to interplay with the immune system in maintaining tissue homeostasis. The strategy of applying a computationally derived immunological score to capture the heterogeneity of the immunological component of normal tissues adds reason to its application as an immunological meta-analysis to cancer transcriptomes. Indeed, quantifying the immunological component of expression studies linked to clinical annotations can lead to informative insights into the immune profiles of patient groups. The necessity and timeliness of applying such a comprehensive computational strategy to tumor expression profiles is highlighted by the increasing reports of immune cell infiltrates in tumor microenvironments as predictors of prognosis and survival in various cancers [4,5,7,36-41].

A proposition for an immunological grading of a tumor based on immune infiltrates has recently been made [42], which would require the expertise of highly trained pathologists. Recent studies in malignant melanoma advocate stratification based on molecular signatures from expression profiling [5,18]. The computational approach described here serves in the automatic identification of ranked immunological signatures and their network of interactions, which leads to a strategy of grading the immunological component of the gene expression of a tumor.

Melanoma was chosen to be the cancer type to demonstrate this strategy, because of the prominent immunological properties of normal skin [43,44] and the strong tendency of melanoma to metastasize [45]. Among the genes harboring some of the highest immunological relevance, and with expression differences in both primary and metastatic profiles compared to normal skin, were the *CD4 *and *CD8 *genes. This indicates that our strategy pinpoints possible recruitment of the adaptive immune response at early points in the progression of melanoma in these tumors, which is interesting in the context of increasing evidence that adaptive immunity influences the behavior of human tumors [36]. With respect to melanoma, this further coincides with recent evidence in mice that the metastatic transition is an early event, and that proliferation of disseminating cells is mediated by the function of CD8^+ ^T-cells [46]. Concerning clinical analysis of metastatic melanoma patients, this approach classified the patient group that had immune signatures of upregulated high immunologically relevant genes, and the proliferative-tumor group with down downregulation of high immunologically relevant genes. It was apparent from the clinical analysis that patients had unique combinations of clinical annotations with both up and downregulated genes with high immunological scores. The distinctive immunological profiles for each patient may reflect the uniqueness of the immune component of each microenvironment and the contradictory role immune genes play in regulating cancer development [47].

This strategy does not grade the directionality of these paradoxical roles in the tumor immune response. Rather, it identifies and grades the magnitude of the immune component of the expression profiles. We propose, however, that improving this strategy to do so will precipitate the characterization of detailed mechanisms underlying tumor-immune surveillance, tolerance and escape and facilitate identification of powerful prognostic factors.

## Conclusions

We have assigned a ranked immunological relevance score to all human genes applying a novel computational approach that utilizes information theory applied to the medical literature. This score was used to chart immunological relevance against the landscape of protein interaction networks. We propose that this approach can be applied to elucidate the phenotypical component of any complex disease. In this study we focus on tumor immunity and melanoma to demonstrate the ability of this strategy to identify and grade the magnitude of the immune component of patient expression profiles. The capability to analyze tumor transcriptional profiles on a genome-wide scale offers a means to investigate the immunological mechanisms of the complex tumor immune relationships. In so doing, such an approach can classify melanoma patient groups into varied immune profiles that correlate with survival and other clinical phenotypes.

## Methods

### Defining the dictionary of terms for immune and neoplasm relevance

By doing manual searches in the Gene Ontology (GO) [48] and the Medical Subject Headings (MeSH) (http://www.nlm.nih.gov/mesh/) resources and documenting those terms deemed relevant for the context, we compiled a list of 1921 immune and 562 neoplasm context terms. This resulted in a comprehensive term list from structured vocabularies that define the contexts in our analysis. The manual searches were implemented using domain knowledge of immunity and cancer. Strict scrutiny of relevance to the context was applied before acceptance of a term into the context term list. The manual searches in MesH and GO produced a candidate list of terms. Each candidate term was read and then categorized as being relevant or not relevant for immunity or cancer based on the expert knowledge of an immunologist or cancer researcher, respectively. As the purpose of this study was to quantify the size of the immune component of tumor samples, a broad scope of immune terms was accepted, each term has an association of an immune function, process, cellular anatomy or immune condition according to the scrutiny of the immunologist. The complete list of chosen immune and neoplasm terms is presented in Additional file 1.

### Extraction of human genes, immune and neoplasm terms from Medline

One of the important elements in the approach is to identify literature co-citations of human genes and their associated GO and MeSH terms by using an established method in text mining [19]. Here is a brief summary of this method with more detail in the referenced article. All official symbols, names and alias symbols for human genes compiled from the Human Genome Organization (HUGO) (http://www.genenames.org/), OMIM (http://www.ncbi.nlm.nih.gov/omim/), and EntrezGene (http://www.ncbi.nlm.nih.gov/gene), were automatically extracted from all Medline article titles and abstracts. The genes are indexed to PubMed IDs after a natural language processing (NLP) step of the Medline abstracts that involves procedures in part of speech tagging (POS) and noun chunking, the purpose of which is to remove false positives of biological term mentions. Some other steps in obtaining the gene citation data of higher quality is to remove abbreviation type false positives, which occur frequently because gene symbols often coincide with other abbreviations having no connection or relevancy with the gene symbol. Such data quality steps yield a greater number unambiguous gene symbol citations in text with an improved precision. In a similar manner to the extractions of gene from Medline text GO terms are extracted using NLP techniques of POS and the GO terms mapped to their corresponding identifiers and indexed against noun chunks in Medline sentences. MeSH terms are indexed to Medline abstracts by using the National Library of Medicine's (NLM) annotations of MeSH terms to articles.

### An immunological and cancer relevance score for all human genes using information theory and text mining

The principle of Shannon's entropy was first tested as a sensible measure of information content applied to gene associations derived from text mining. This was further refined using the Kullback-Leibler (KL) score, thus correcting for bias introduced by the popularity of the gene to be co-cited in all of Medline which we found to inherent in the Shannon entropy calculations.

In these information theory aproach we interpreted gene co-citation events in medical articles with terms from a lexicon of expertly chosen annotations from a context as an information coding system for the context (the context of immunity and cancer in this study). The frequency of co-citation events of a manually annotated context term *i *extracted from Medline abstracts using text mining and co-cited with a human gene *x *is treated as a message. This message is detected within each element of an alphabet of symbols of size *N*, where *N *is the total number of annotated terms in the lexicon of that context. Immune and cancer experts manually chose the elements of the alphabet of symbols *N *from the structured biological vocabularies of GO and MeSH. Thus we view the literature association between a gene and a context term as the observance of a symbol describing an element of that contextual message and the probability of that event occurring is:

g_*i *_is the number of co-citations for a gene with a context term *i *in Medline and *iT*_*g *_is the total number of times the context term *i *is cited with all human genes in all of Medline (the total gene co-citation space of the context term). Hence, the continuously expanding 20 million articles of Medline is the source emitting these symbols with probabilities (p1, p2, . . ., p*N*) and these are the symbols of communication that define an immunological (or other contextual) score for all human genes. We assume that the symbols are emitted independently for each gene. In this assumption the probability that a gene is associated to, for example, the immune term "T-cell differentiation" in Medline is independent of its association to the immune term "Macrophage" and their probabilities are computed independently. These probabilities of events (p1, p2, . . ., p*N*) give discrete values that can be used to detect the size of a message and thus the contextual information content for each gene as defined by Shannon's entropy [49]

Although the Shannon entropy provided the accurate size of the information content for each gene, it did not account for bias introduced by the popularity of the gene Medline. We therefore refined the information theory approach to correct for this bias. This bias was defined as the popularity of the gene to be co-cited in all of Medline, *i.e. *its probability of co-citation among all GO and MeSH terms in the gene co-citation space of Medline. We quantified this bias and corrected for it using the Kullback-Leibler (KL or "relative entropy") calculation to create a more accurate measure of information content that can be used as the immunological and cancer score for each gene. The KL was used to determine the divergence of the observed probability *p(x)*, described above, from an assumed incorrect distribution, which we take as the popularity of the gene in the total Medline gene co-citation space *q(x):*

Where *g*_*T *_is the number of co-citations for a gene with all GO and MeSH terms in Medline and *GS*_*T *_is the total number of co-citation events for all GO and MeSH terms with all human genes in all of Medline (the total gene co-citation space of GO and MeSH, the source of the immune and cancer context terms chosen by domain experts). This measures the expected amount of information required to code a message from a context term for a gene *p(x) *when using a code based on the assumed incorrect probability *q(x) *rather than using a code based on *p(x) *and is defined by Kulback-Leibler KL as:

As this relative entropy score (KL) corrects for the bias *q(x) *for each gene, it was used as to calculate the "immunological and cancer relevance" score throughout this study.

### Collating manually curated immune relevant gene sets

The immunology gene sets were compiled from the following manually curated sources: (1) Immport (https://www.immport.org), (2) Immunome [20], (3) Iris [31], (4) Mapk-Nfkb (ref), (5) Septic Shock (http://www.septicshock.org) and (6) InnateDB [30]. The HUGO (http://www.genenames.org/) symbol for genes provides a unique identifier for human genes and is ideal for the integration of text mining derived knowledge. It was used in this study to integrate and determine the overlapping descriptive statistics for each of the six databases and visualized in Venn diagrams in the VennMaster software [50] to approximate their intersections by incorporating the gene set size information. Similarly genes from two efforts to catalogue the inflammatory response [21,22] were integrated using their HUGO gene symbols and compared to the unified immune gene set from the above six different sources mentioned above.

### Constructing a validated human interactome & network analysis

We constructed a human gene network by integrating binary human interactions from IntAct [51], BioGRID [51] and HPRD [52]. Each of these datasets of binary interacting protein pairs was downloaded from their source and the unique ids of the interactors were cross-referenced to their NCBI gene IDs and official Gene Symbols. This resulted in a unified set of binary NCBI gene ID interactor pairs, with their corresponding official gene symbols. The interaction data was limited to these sources as they consist of validated protein-protein interactions with experimental evidence curated from critical reading of the scientific literature by expert biologists.

This integrated data set is represented as an undirected, unweighted network, where G = (V,E) comprising of a set of nodes V and edges E. Each node represents a human gene and each edge represents a pair of genes (u,v) as a representation of a binary interaction in the human interactome. If there exists a physical binary interaction between u and v, in at least one of the protein products of each gene, an edge is connected. The tissue specific interactomes were derived from the entries in the three protein interaction databases mentioned above and the tissue expression annotations from in a recent study integrating tissue specific interactions from 79 human tissues [25].

Network centrality analysis was carried out on the networks by means of calculating five measures of centrality for each gene in the interactome (Connectivity, betweeness, eccentricity, closeness and eigenvector). A descriptions of equations implemented for these measures and full details of their context to protein networks in cancer are summarized here [53]

### Microarray gene expression analysis and a composite expression and immunological relevance score

Tissue specific gene expression data from the Symatlas project [24] was analyzed to detect pairwise differential expression across the 79 specific tissues [25] (Additional file 8). We considered a gene differentially expressed between any pair of tissues and therefore viable for further analysis if there was greater than a two times fold-change in expression. The average immunological score was then determined for these differentially expressed genes across all tissue pairs. A similar approach was used for profiles in the progressive states of skin cancer. For the gene expression profile linked to patient survival probes [18] strict criteria were applied to reduce false positive signals in that only those probes with detection p-value < 0.01 in more than 50 out of the total 57 patients were used. The software used to calculate the detection p-values (Illumina BeadStudio) uses a nonparametric method for the computation of detection p-values. In this method the z-values of the probe signals are ranked relative to the z-values of the negative control signals. These were quantile normalized [54], and log2 transformed. Each probe signal intensity measurement (S) was given a fold change relative to that probes mean signal intensity (MSI) across all patients (P) and utilized to create a weighed composite signal intensity and immunological score for each gene (W_g_):

The weighted expression and immune score for each gene was then summated across all genes (M) for each patient to generated a weighted immune score for each patient (W_p_):

The patient scores where compared to the clinical annotations to find correlations between the weighted immunological score (W_p_) and the clinical phenotypes. Monte Carlo simulations with 10,000 draws were used to create a null distribution for each comparison. For numerical phenotypes Pearson's correlation were used.

## Competing interests

The authors declare that they have no competing interests.

## Authors' contributions

TC conceived and developed the information theory approach to quantify the immune and cancer components. TC, TJL, and EH designed and planned the study. MP, DS, FC and TC performed the network analysis. VN performed the gene expression profiling. EH, TJL, MB, and TC applied and developed the manual curation pipeline and biological interpretation of the data. TC drafted the manuscript. EH, TC and TJL wrote the final manuscript. All authors read and approved the final manuscript.

## Pre-publication history

The pre-publication history for this paper can be accessed here:

http://www.biomedcentral.com/1755-8794/4/28/prepub

## Supplementary Material

Additional file 1 **Tables of manually curated immune and neoplasia terms**. These are the terms used to define the dictionary of terms for immune and neoplasm context. Manually selected from GO and MeSH using domain knowledge. Click here for file

Additional file 2 **Genome-wide ranked Immunological and neoplasia relevance score for genes**. Table depicting the immunological and cancer relevance score for all human genes quantified in bits using information theory calculation with Kullback-Leibler adjustments Click here for file

Additional file 3 **Immunological relevance of non-curated genes**. Ranked immunological relevance of genes not populated in the manually curated immune gene resources Click here for file

Additional file 4 **Relationship of both immunological and cancer relevance to network centrality**. Tables reporting the Pearson correlation coefficients of immunological and cancer relevance to the principle network centrality measures of the human interactome. Click here for file

Additional file 5 **Tumor immunity interactome landscape**. The underlying data behind Figure 3, quantifying in bits of information the immunological and cancer relevance charted against connectivity in the interacome Click here for file

Additional file 6 **Table of the k-means classification by means of the eccentricity centrality measure, showing biologically meaningful classes of tissues**. K-means classification of tissue groups shown in Figure 5 (parameter K = 9). Determined by means of the eccentricity centrality measure for each of the tissue specific interactomes from the SymAtlas [24]. Click here for file

Additional file 7 **Bogunovic et al, 2009 distinct patient profiles and relationship to clinical phenotypes**. Composite expression and immunological relevance score for all genes in each patient in this study. Demonstrated here as an example to offer an overview of the diversity and uniqueness of the immunological profile, detected by this approach in each individual patient samples. Click here for file

Additional file 8 **Normal tissue index**. Index for the 79 normal tissues from the SymAtlas [24] depicted in the heatmap of immunological comparisons in Figure 4A Click here for file
