# Supplementary material for: Immunological network signatures of cancer progression and survival
Source: BMC Med Genomics. 2011 Mar 31;4:28. doi: 10.1186/1755-8794-4-28 (PMC3094196; doi:10.1186/1755-8794-4-28)
Supplement: Additional file 6 — Table of the k-means classification by means of the eccentricity centrality measure, showing biologically meaningful classes of tissues. K-means classification of tissue groups shown in Figure 5 (parameter K = 9). Determined by means of the eccentricity centrality measure for each of the tissue specific interactomes from the SymAtlas [24]. [file 1755-8794-4-28-S6.PDF]

Cluster number	tissue network
1	BM_CD105_Endothelial
1	BM_CD33_Myeloid
1	BM_CD34_
1	PB_BDCA4_Dentritic_Cells
1	PB_CD14_Monocytes
1	PB_CD19_Bcells
1	PB_CD56_NKCells
2	TestisGermCell
2	TestisInterstitial
2	TestisLeydigCell
2	TestisSeminiferousTubule
2	testis
3	CerebellumPeduncles
3	CingulateCortex
3	MedullaOblongata
3	OccipitalLobe
3	ParietalLobe
3	Pons
3	TemporalLobe
3	caudatenucleus
3	cerebellum
3	globuspallidus
3	subthalamicnucleus
4	Heart
4	Liver
4	Lung
4	PLACENTA
4	adrenalgland
4	bonemarrow
4	kidney
4	thymus
5	Amygdala
5	Hypothalamus
5	Pituitary
5	PrefrontalCortex
5	Thalamus
5	WholeBrain
5	fetalbrain
5	spinalcord

Cluster number	tissue network
6	721_B_lymphoblasts
7	BM_CD71_EarlyErythroid
7	ColorectalAdenocarcinoma
7	bronchialepithelialcells
7	leukemiachronicmyelogenous_k562_
7	leukemialymphoblastic_molt4_
7	leukemiapromyelocytic_hl60_
7	lymphomaburkittsDaudi
7	lymphomaburkittsRaji
8	Appendix
8	DRG
8	Ovary
8	SkeletalMuscle
8	SuperiorCervicalGanglion
8	TrigeminalGanglion
8	atrioventricularnode
8	ciliaryganglion
8	skin
9	ADIPOCYTE
9	AdrenalCortex
9	CardiacMyocytes
9	OlfactoryBulb
9	Pancreas
9	PancreaticIslets
9	Prostate
9	SmoothMuscle
9	TONGUE
9	Thyroid
9	Tonsil
9	Uterus
9	UterusCorpus
9	fetalThyroid
9	fetalliver
9	fetallung
9	lymphnode
9	salivarygland
9	trachea
10	PB_CD4_Tcells
10	PB_CD8_Tcells
10	WHOLEBLOOD
